# Establishment and application of a quadruple RT-qPCR method for simultaneous detection of porcine enteric coronaviruses

**DOI:** 10.3389/fvets.2025.1714780

**Published:** 2025-12-10

**Authors:** Caiwang Ye, Jingru Xu, Sisi Fan, Fangting Chen, Shuqi Qiu, Huachun Pan, Shumin Yang, Yuqi Li, Yuting Liao, Weiye Lin, Xiaoziyi Xiao, Xuejin Li, Yuxi Xue, Yali Kang, Yubin Zhuo, Lingshan Huang, Xiaoping Wu, Ailing Dai, Nana Yan, Kewei Fan

**Affiliations:** 1 Fujian Provincial Key Laboratory for Prevention and Control of Animal Infectious Diseases and Biotechnology, College of Life Sciences, Longyan University, Longyan, China; 2 Agriculture and Rural Affairs Bureau of Nanping City, Fujian, China; 3 College of Animal Science, Fujian Agriculture and Forestry University, Fujian, China

**Keywords:** porcine enteric coronaviruses, porcine epidemic diarrhea virus (PEDV), porcine transmissible gastroenteritis virus (TGEV), porcine deltacorona virus (PDCoV), porcine enteric alphacoronavirus (PEAV), RT-qPCR

## Abstract

**Introduction:**

Porcine enteric coronaviruses (PECs) are a group of viruses that cause severe diarrhea in piglets, significantly impacting the pig industry and resulting in huge economic losses. Important PECs include porcine epidemic diarrhea virus (PEDV), porcine enteric alphacoronavirus (PEAV), porcine deltacoronavirus (PDCoV), and transmissible gastroenteritis virus (TGEV). These pathogens cause highly similar clinical symptoms and pathological changes in piglets. Therefore, it is necessary to develop a simultaneous detection method for the precise prevention and control of porcine diarrheal diseases.

**Methods:**

To establish a rapid, simple, and accurate detection method for differential diagnosis on these four pathogens, this study designed specific primers and probes based on the conserved regions of the M gene of PEDV, PEAV, and PDCoV, and the N gene of TGEV. By optimizing the reaction conditions, a quadruple fluorescence real-time quantitative RT-PCR (RT-qPCR) method for simultaneous detection of the four porcine diarrhea viruses was developed.

**Results:**

This method demonstrated high specificity, with no cross-reactivity with other common porcine pathogens such as porcine reproductive and respiratory syndrome virus, porcine circovirus, classical swine fever virus, porcine rotavirus, and pseudorabies virus. The sensitivity was excellent, with the limit of detection of 100 copies/μL in multiplex real-time RT-qPCR assays and all correlation coefficients (R2) exceeding 0.99. Repeatability was also strong, with the coefficient of variation of the intra- and inter-assay repeatability tests ranging from 0.3% to 1.0%.When applied to 231 clinical samples from Fujian province, the multiplex RT-qPCR method identified PEDV as the predominant pathogen, and often in co-infections. These results were 100% consistent with those from the commercial RT-qPCR kits, demonstrating the high accuracy of the developed method.

**Discussion:**

In summary, this study established a specific, sensitive, and accurate multiplex RT-qPCR assay for the simultaneous detection of PEDV, PEAV, TGEV, and PDCoV, providing a valuable tool for the monitoring and differential diagnosis of these four PECs.

## 1 Introduction

In recent years, porcine diarrheal diseases have become common on pig farms in China, causing significant economic losses to the pig industry (1). Porcine enteric coronaviruses (PECs), including porcine epidemic diarrhea virus (PEDV), porcine enteric alphacoronavirus (PEAV), porcine deltacoronavirus (PDCoV), and transmissible gastroenteritis virus (TGEV), are the primary pathogens responsible for these outbreaks. They cause intestinal diseases, watery diarrhea, and high mortality in infected piglets (1). Consequently, the prevalence of PECs poses a major threat to the pig industry in China and worldwide, a concern heightened by the risk of cross-species transmission (1).

PECs are large, single-stranded, positive-sense RNA viruses belonging to the *Coronaviridae* family and order *Nidovirales* (2). PEDV and TGEV cause highly similar clinical symptoms, including diarrhea, vomiting, anorexia, dehydration, and weight loss in piglets (3). PEDV was first isolated and identified in the UK in 1978 (4) and has since spread globally, causing ongoing economic losses (5). TGEV was first reported in the United States in 1946, and then it was detected in Americas, Asia, and Europe, showing its ability to spread across countries (5, 6). It is most severe in piglets under 2 weeks of age, with a mortality rate approaching 100% (5). PDCoV, a member of the genus *Deltacoronavirus*, is an enveloped virus with a single-stranded, positive-sense RNA genome and characteristic spike proteins on its surface (2). The first major PDCoV outbreak occurred in the US (7), and the virus has now been detected in many countries, including China, South Korea, Canada, Japan, Mexico, and Thailand, posing a serious threat to the global pig industry (2). Moreover, many other species have been confirmed to be susceptible to PDCoV infection, including calves, chickens, turkey poults, and mice (2). A report of human infection in Haitian children (8) underscores its potential for cross-species transmission and the associated threat to public health. PEAV, which belongs to the genus *Alphacoronaviruses*, was first reported in China in 2017 (9). The clinical symptoms it causes are extremely similar to those of other PECs (5), which often leads to its being overlooked or misdiagnosed, thereby increasing the threat to herd health.

These four PECs are common pathogens that cause fatal diarrheal syndrome in newborn piglets. Co-infections are frequent and present a more complex clinical situation than single infections, making accurate diagnosis, targeted prevention, and effective treatment challenging (10). Therefore, developing a method for the simultaneous detection of these viruses is necessary for the precise prevention and control of porcine diarrheal diseases. Multiplex fluorescence real-time quantitative PCR (RT-qPCR) is a high-throughput technique that allows for the simultaneous detection of multiple pathogens in a single reaction, featuring high sensitivity, high efficiency, and high specificity (11). Compared to traditional diagnostic methods for detecting these four PECs, such as conventional RT-PCR and singleplex RT-qPCR, multiplex RT-qPCR can significantly shorten the diagnostic time and enable precise and early diagnosis of co-infections (12). Previous studies have reported three assays for the detection and differentiation of PEDV, PEAV, PDCoV, and TGEV (13–15). The first assay used PEDV M (membrane), PEAV N (nucleocapsid), PDCoV M, and TGEV N genes as target templates. The second used PEDV N, PEAV N, PDCoV N, and TGEV S genes, and the third used PEDV N, PEAV N, PDCoV M, and TGEV M genes (Table 1). In this study, we developed a multiplex real-time RT-qPCR assay to simultaneously detect all four viruses. We designed specific primers and probes for conserved regions of the M gene of PEDV, PEAV, and PDCoV, and the N gene of TGEV. After optimizing the reaction conditions, we rigorously evaluated the assay's sensitivity, specificity, and repeatability. When applied to a wide range of clinical samples, including feces, intestinal tissue, and anal swabs, this method proved effective for the rapid and precise detection of these four viruses.

**Table 1:** Comparison with previous reports on quadruple RT-qPCR for the detection of PEDV, PEAV, PDCoV, and TGEV.

**Study**	**Target templates**	**Sensitivity**	**Clinical sample types**
(13)	PEDV M, PEAV N, PDCoV M, and TGEV N genes	1.1 × 10^2^ copies/μL	Fecal samples
(14)	PEDV N, PEAV N, PDCoV N, and TGEV S genes	8 genomic copies/reaction for PEDV; 6.8 genomic copies/reaction for PEAV; 4 genomic copies/reaction for PDCoV; 16 genomic copies/reaction for TGEV.	Fecal samples
(15)	PEDV N, PEAV N, PDCoV M, and TGEV M genes	1.1 × 10^2^ copies/μL	Fecal samples
This study	PEDV M, PEAV M, PDCoV M, and TGEV N genes	1.0 × 10^2^ copies/μL	Feces, intestinal tissue, and anal swabs

## 2 Materials and methods

### 2.1 Viruses and clinical samples

PEDV, PEAV, PDCoV, TGEV, porcine reproductive and respiratory syndrome (PRRSV), porcine circovirus (PCV), classical swine fever virus (CSFV), porcine rotavirus (PoRV), and pseudorabies virus (PRV) were provided and stored in the Fujian Provincial Key Laboratory for Prevention and Control of Livestock Infectious Diseases and Biotechnology. A total of 231 clinical diarrheal samples including 74 intestinal samples, 92 fecal samples, and 65 anal swabs were collected from large-scale pig farms in different regions in Fujian between 2016 and 2022.

### 2.2 Primers and probes design

Based on the sequence of the conserved regions of M gene of PEDV, PEAV, and PDCoV strains and N gene of a TGEV strain recorded in GenBank (accession number: JX188454.1, MF167434.1, KP757890.1, and KX900409.1, respectively), four pairs of specific primers and *TaqMan* probes were designed using Oligo 7.37 (Table 2). Both primers and probes were synthesized by ShangHai Sangon Biotech (Shanghai, China).

**Table 2:** Primer and probe information and the components of reaction system for multiple RT-qPCR.

**Pathogens**	**Primers/probes**	**Sequence (5^′^-3^′^)**	**Product size (bp)**	**Reaction volume (10 pmol/μL)**	**Other components**
PEDV	PEDV-F	CCTGAAACAGACGCGCTTCT	180	0.5 μL	2 × Animal Detection U Probe Master Mix 12.5 μL; cDNA template 2 μL; ddH2O 9.3 μL
	PEDV-R	CTTGGCGACTGTGACGAAATT		0.5 μL	
	PEDV-P	FAM-ACTACTTCTGTGATGGGC-BHQ1		0.2 μL	
PEAV	PEAV-F	CATGCCAGTCCAGGCCTCAA	120	0.5 μL	2 × Animal Detection U Probe Master Mix 12.5 μL; cDNA template 2 μL; ddH2O 9.3 μL
	PEAV-R	CACGCTTCCATTCAGGTTTGT		0.5 μL	
	PEAV-P	HEX-TGGTAAAAATACACCCAAACC-BHQ1		0.2 μL	
PDCoV	PDCoV-F	TGGGTACATGGAGGTGCATTC	135	0.5 μL	2 × Animal Detection U Probe Master Mix 12.5 μL; cDNA template 2 μL; ddH2O 9 μL
	PDCoV-R	CCATATCCTGTGGCGGATTT		0.5 μL	
	PDCoV-P	Cy5-CATCGACCACATGGC-BHQ2		0.5 μL	
TGEV	TGEV-F	GGCCAACGTAAAGAGCTTCCT	143	0.5 μL	2 × Animal Detection U Probe Master Mix 12.5 μL; cDNA template 2 μL; ddH2O 9 μL
	TGEV-R	CCAAGCGTGGTTGGTTTGTT		0.5 μL	
	TGEV-P	ROX-AAAGGTGGTTCTTCTACTACTTA-BHQ3		0.5 μL	

### 2.3 Construction and identification of the standard plasmids

Sample RNA of PEDV, PEAV, PDCoV, and TGEV was extracted using the FastPure Viral DNA/RNA Mini Kit V2 (Vazyme Biotech, China), and then was reverse-transcribed to cDNA using HiScript III 1st Strand cDNA Synthesis Kit (Vazyme Biotech, China) and used as the templates. M gene and N gene were amplified using specific primers in Table 1, and the amplification products were ligated into the pMD19-T vector (Takara Biotechnology, China). The recombinant plasmids were then transformed into DH5α competent cells (Shanghai Weidi Biotech, China). Single-positive colonies were selected, cultured, and sent to Shanghai Sangon Biotechnology for sequencing. The correctly sequenced plasmids were designated as pPEDV-M, pPEAV-M, pPDCoV-M, and pTGEV-N, respectively. Plasmids were extracted using FastPure Plasmid Mini Kit (Vazyme Biotech, China). The concentration of the recombination plasmids was determined using a NanoDrop One ultramicrospectrophotometer (ThermoFisherScientific, Shanghai, China), and the copy number was calculated according to the formula: plasmid (copies/μL) = (6.02 × 10^23^) × (plasmid concentration) (ng/μL) × 10^−9^/(plasmid length × 660) (16).

### 2.4 Optimization of reaction conditions

Four standard plasmids of pPEDV-M, pPEAV-M, pPDCoV-M, and pTGEV-N were mixed and used as templates. The reaction system was prepared according to instructions of 2 × Animal Detection U Probe Master Mix (Vazyme Biotech, China). The optimal reaction conditions for multiple RT-qPCR were obtained by optimizing the primer concentration (final concentration of 0.1–0.8 pmol/μL), probe concentration (final concentration of 0.1–0.8 pmol/μL), and annealing temperature (58 °C−64 °C) using the control variate method.

### 2.5 Establishment of the standard curves

The four standard plasmids mixed in equal volume were diluted in a 10-fold series serial, resulting in a range of concentrations from 10^2^ to 10^7^ copies/μL. Real-time fluorescent quantitative RT-qPCR amplification of the diluted standard plasmids was performed in triplicate for each dilution using the optimized reaction system and protocol, and the standard curve was established. The logarithm of the initial copy number of the standard plasmid DNA is plotted on the *X*-axis, whereas the cycle threshold (Ct) values obtained from real-time fluorescent quantitative PCR are plotted on the *Y*-axis.

### 2.6 Specificity test

The specificity of our multiplex RT-qPCR system was evaluated using the optimized conditions and system with DNA/cDNA templates from PEDV, PEAV, PDCoV, TGEV, PRRSV, PCV, CSFV, PoRV, and PRV. The standard plasmids and sterile double-distilled water (ddH_2_O) were used as the positive control and negative control, respectively. All templates were verified by PCR or viral isolation methods to confirm their identity. All reactions were conducted in triplicates.

### 2.7 Sensitivity test

Four standard plasmids mixed in equal volume were diluted in a 10-fold series, and the resulted templates of 10^1^ to 10^9^ copies/μL were used as templates. The sensitivity of our system was determined by amplifying various dilutions of the templates with this optimized reaction system. ddH_2_O was used as the negative control. All reactions were conducted in triplicates.

### 2.8 Repeatability test

Four standard plasmids mixed in equal volume were diluted in a 10-fold series, and the resulted dilutions of 10^2^, 10^4^, and 10^6^ copies/μL were selected as templates. Intra repeatability tests were conducted by preparing three replicates for each dilution, and inter repeatability tests were conducted by preparing the reaction reagents three times at different times. The average coefficient of variation (CV) was calculated according to the formula: CV (%) = (standard deviation/mean) × 100%, and it was calculated in Excel. All reactions were conducted in triplicates.

### 2.9 Clinical sample testing

A total of 293 clinical samples were tested. One milliliter PBS was added to 10 mg of the sample. Next, the intestinal samples were grounded in a grinder and then centrifugated at 5,000 rpm for 5min; the fecal samples and anal swabs were repeatedly twice and then centrifugated at 5,000 rpm for 10 min. Two hundred microliter of the supernatants were taken to extract RNA, followed by reverse-transcription to obtain cDNA as the templates and tested using the multiplex RT-qPCR developed in this study. Meanwhile, the commercialized VetMAX PEDV/TGEV/SDCoV real time RT-PCR Kit (ThermoFisher Scientific, China) and the Porcine Acute Diarrhea Syndrome Coronavirus Fluorescence Quantitative RT-PCR Kit (15-84900y, Yaji Biological, China; research only) were used to test above samples. The detection results of the method established in this study were compared with those of the above method, and the coincidence rate was calculated.

## 3 Results

### 3.1 Construction and identification of the standard plasmids

The M gene of PEDV, PEAV and PDCoV, and the N gene of TGEV were amplified from their cDNA and ligated into pMD19-T vectors and sent for sequencing. The plasmids with correct sequence of insertions were designated as pPEDV-M, pPEAV-M, pPDCoV-M, and pTGEV-N, respectively, and used as the standard plasmids. The concentration of above plasmids was determined using a ultramicrospectrophotometer: 122.6 ng/μL for pPEDV-M, 88.3 ng/μL for pPEAV-M, 120.8 ng/μL for pPDCoV-M, and 102.7 ng/μL pTGEV-N. The copy number were calculated according to the formula (16):


$$\begin{array}{r} \text{copy}\;\text{number}\;\text{(copies/}\mu \text{L)}=\\ \frac{\text{Concentration}\;\text{(ng/}\mu \text{L)}\times 10^{-9}\times 6.022\times 10^{23}}{\text{DNA}\;\text{length}\times 660} \end{array}$$ (1)

The copy number of these standard plasmids were calculated to be: 3.63 × 10^10^ copies/μL for pPEDV-M, 2.67 × 10^10^ copies/μL for pPEAV-M, 3.63 × 10^10^ copies/μL for pPDCoV-M, and 3.08 × 10^10^ copies/μL for pTGEV-N. These plasmids were mixed in equal volume and diluted to 10^10^ copies/μL.

### 3.2 Reaction system procedure and optimization

The quadruple RT-qPCR procedure was optimized using control variate method using the standard plasmids as the templates. The results of the optimal reaction were determined as follows: 2 × Animal Detection U Probe Master Mix 12.5 μL, probes (10 pmol/μL) of PEDV, PEAV, PDCoV, and TGEV were 0.2, 0.2, 0.5, and 0.5, respectively, and primers (10 pmol/μL) were 0.5 μL for each virus; 2 μL of the cDNA template and ddH_2_O were added to a final volume of 25 μL (Table 1). All reactions were performed on a LightCycler 480 II real-time fluorescence quantitative PCR instrument (Roche, USA). The optimal amplification program was optimized as follows: 45 cycles of 95 °C for 30 s, 95 °C for 10 s, and 60 °C for 10 s.

### 3.3 Establishment of the standard curves

**Figure 1:**
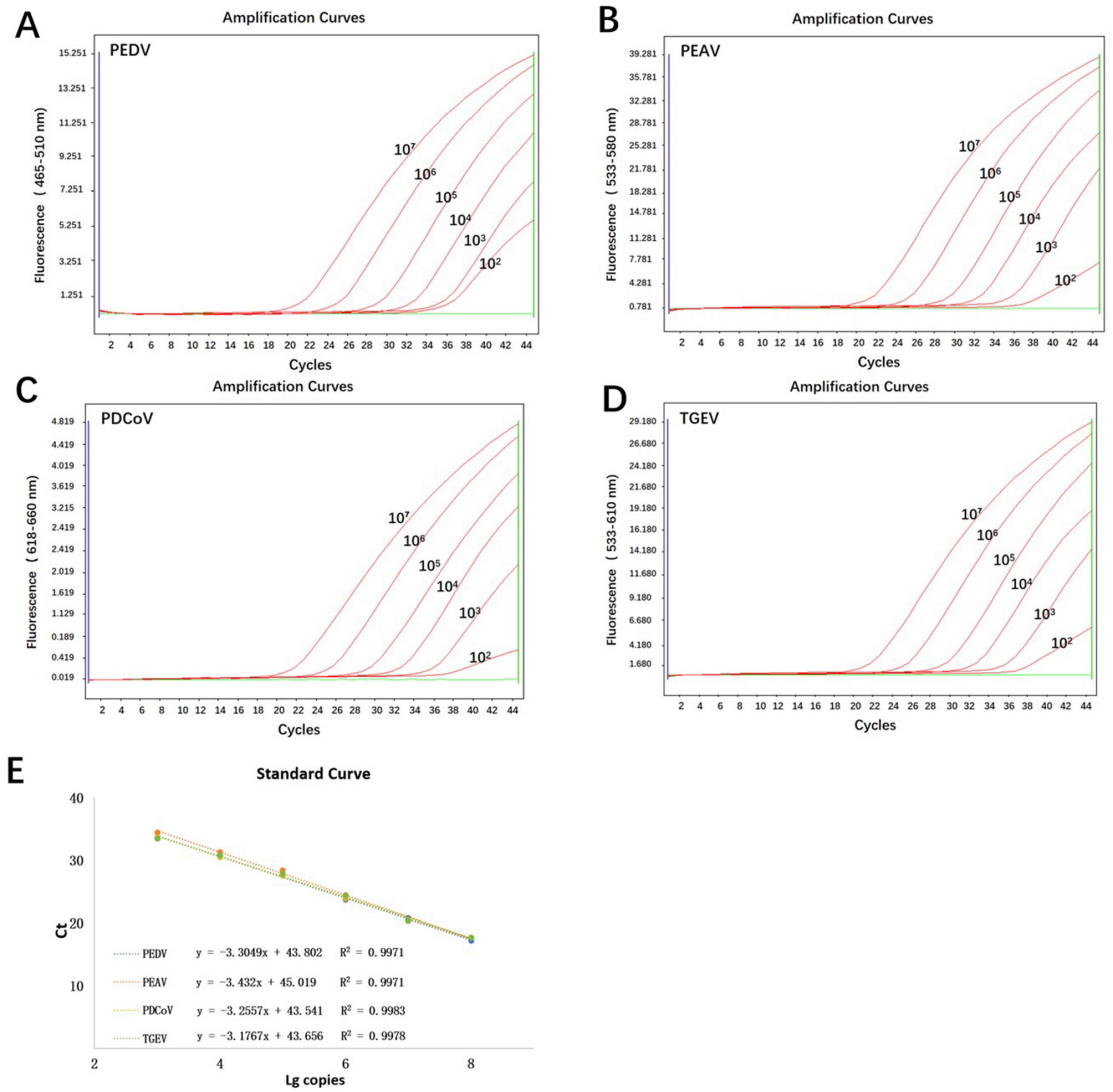
Standard curves of quadruple RT-qPCR based on the amplification curves. **(A–D)** The amplification curves of pPEDV-M, pPEAV-M, pPDCoV-M, and pTGEV-N, respectively; amplification curves: 10^2^-10^7^ copies/μL for the mixture of four plasmid standards; negative control: ddH_2_O; **(E)** The standard curves of pPEDV-M, pPEAV-M, pPDCoV-M, and pTGEV-N, respectively.

Standard curves were established based on the amplification results of real time fluorescence RT-qPCR on standard plasmids, ranging from 10^2^ to 10^7^ copies/μL. Results showed a good amplification efficiency, with good linear relationships between log starting quantity (*X*-axis) and Ct (*Y*-axis). Their linear regression equations are listed as follows: *y* = −3.3049*x* + 43.802, *R*^2^ = 0.9971 (PEDV); *y* = −3.432*x* + 45.019, *R*^2^ = 0.9971 (PEAV); *y* = −3.2557*x* + 43.541, *R*^2^ = 0.9983 (PDCoV); *y* = −3.1767*x* + 11.513, *R*^2^ = 0.9978 (TGEV); (Figure 1).

### 3.4 Specificity analysis

**Figure 2:**
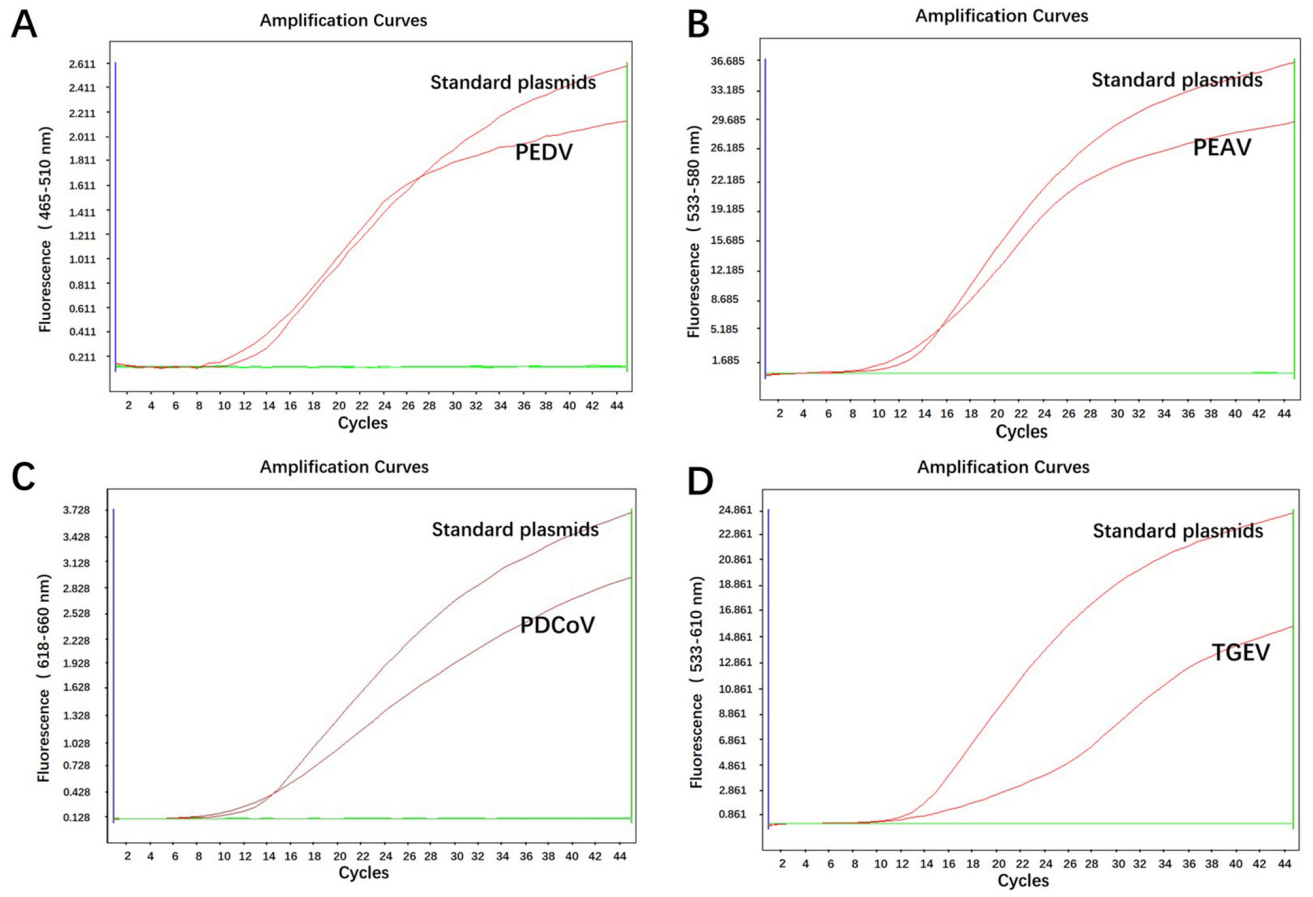
Specificity of the quadruple RT-qPCR. **(A–D)** Specificity detection for PEDV, PEAV, PDCOV, and TGEV.

The DNA/cDNA templates from PEDV, PEAV, PDCoV, TGEV, PRRSV, PCV, CSFV, PoRV, and PRV were amplified using the optimized quadruple RT-qPCR system. Results showed that only PEDV, PEAV, PDCoV, TGEV, and the standard plasmids exhibited fluorescence signals, and neither fluorescence signals nor amplification curves were observed in the wells containing other interfering DNA/cDNA or negative controls (Figure 2). This demonstrates the high specificity of our quadruple RT-qPCR method.

### 3.5 Sensitivity analysis

**Figure 3:**
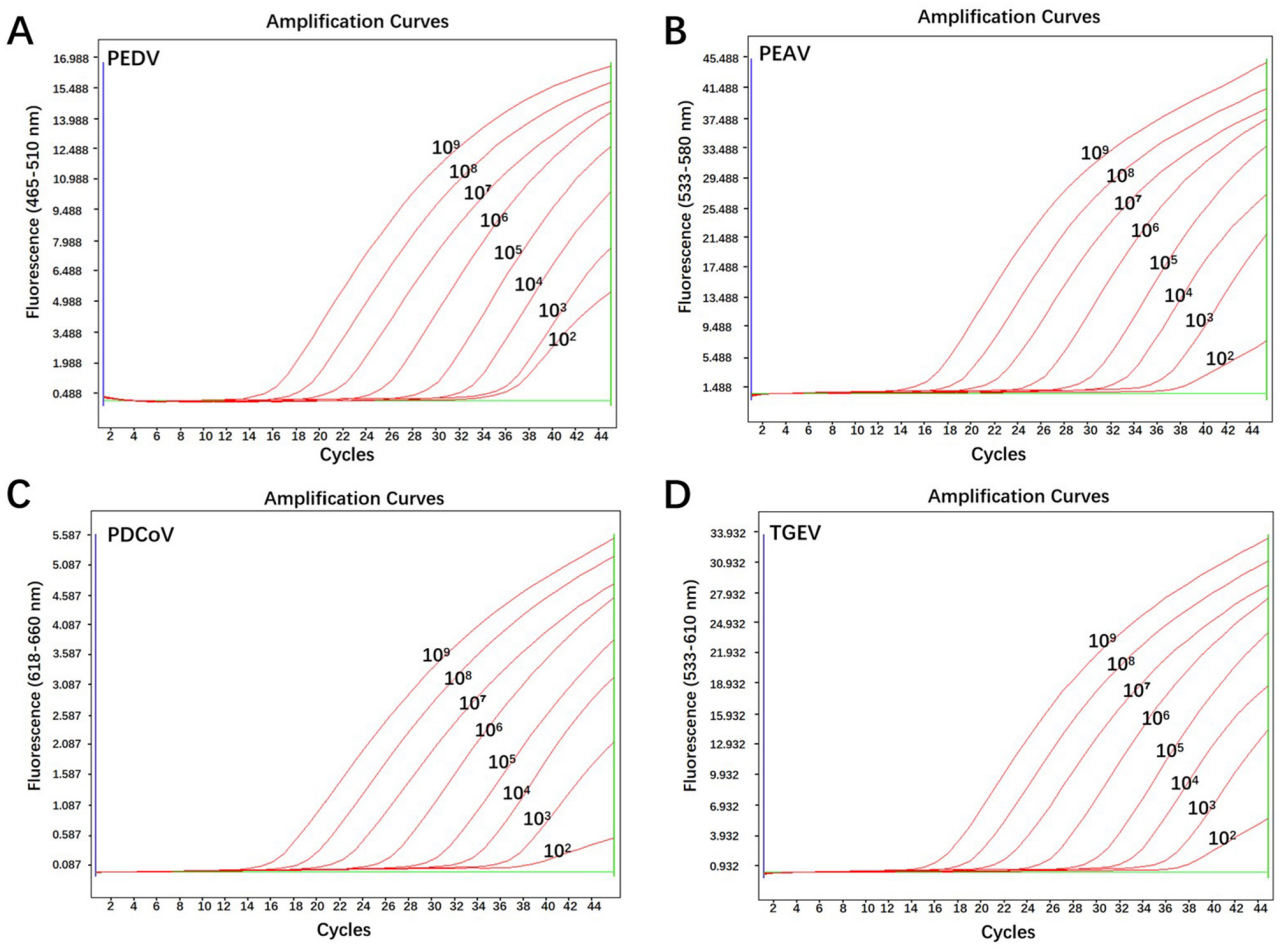
Sensitivity analysis of the quadruple RT-qPCR. **(A–D)** Amplification curves of pPEDV-M, pPEAV-M, pPDCoV-M, and pTGEV-N, respectively; amplification curves: 10^1^-10^9^ copies/μL for the mixture of four plasmid standards; negative control: ddH_2_O.

Sensitivity tests were performed using the mixture of four standard plasmids ranging from 10^1^ to 10^9^ copies/μL as templates. Results showed that the lowest detectable levels of PEDV, PEAV, PDCoV, and TGEV were determined to be approximately 100 copies/μL, respectively (Figure 3), demonstrating the high sensitivity of this quadruple RT-qPCR method.

### 3.6 Repeatability analysis

The repeatability of the quadruplex RT-qPCR was determined by evaluating CVs of the intra-assay and inter-assay. Repeatability tests of three intra- and inter-assays were performed using the mixture of four standard plasmids of 10^2^, 10^4^, and 10^6^ copies/μL, respectively. Results showed that CVs of the intra- and inter-assays were between 0.3 and 1.0% (Table 3), demonstrating the high repeatability and stability of this method.

**Table 3:** Intra- and inter-assay reproducibility of the quadruple RT-PCR assay.

**Plasmids**	**Intra-assay**			**Inter-assay**		
	**x**	**s**	**CV%**	**x**	**s**	**CV%**
**pPEDV-M (copies/**μ**L)**						
10^6^	17.60	0.09	0.5	17.90	0.17	1.0
10^4^	24.51	0.16	0.6	24.61	0.13	0.5
10^2^	30.61	0.30	1.0	30.84	0.20	0.6
**pPEAV-M (copies/**μ**L)**						
10^6^	17.64	0.10	0.5	17.71	0.19	1.0
10^4^	24.47	0.20	0.8	24.90	0.11	0.4
10^2^	31.20	0.21	0.6	31.12	0.25	0.8
**pPDCoV-M (copies/**μ**L)**						
10^6^	17.84	0.10	0.5	18.10	0.12	0.7
10^4^	24.73	0.15	0.6	24.80	0.11	0.4
10^2^	31.60	0.18	0.6	31.75	0.12	0.4
**pTGEV-N (copies/**μ**L)**						
10^6^	17.73	0.07	0.3	17.90	0.15	0.8
10^4^	24.53	0.12	0.5	24.62	0.20	0.8
10^2^	31.65	0.16	0.5	31.81	0.22	0.7

### 3.7 Clinical sample testing

**Figure 4:**
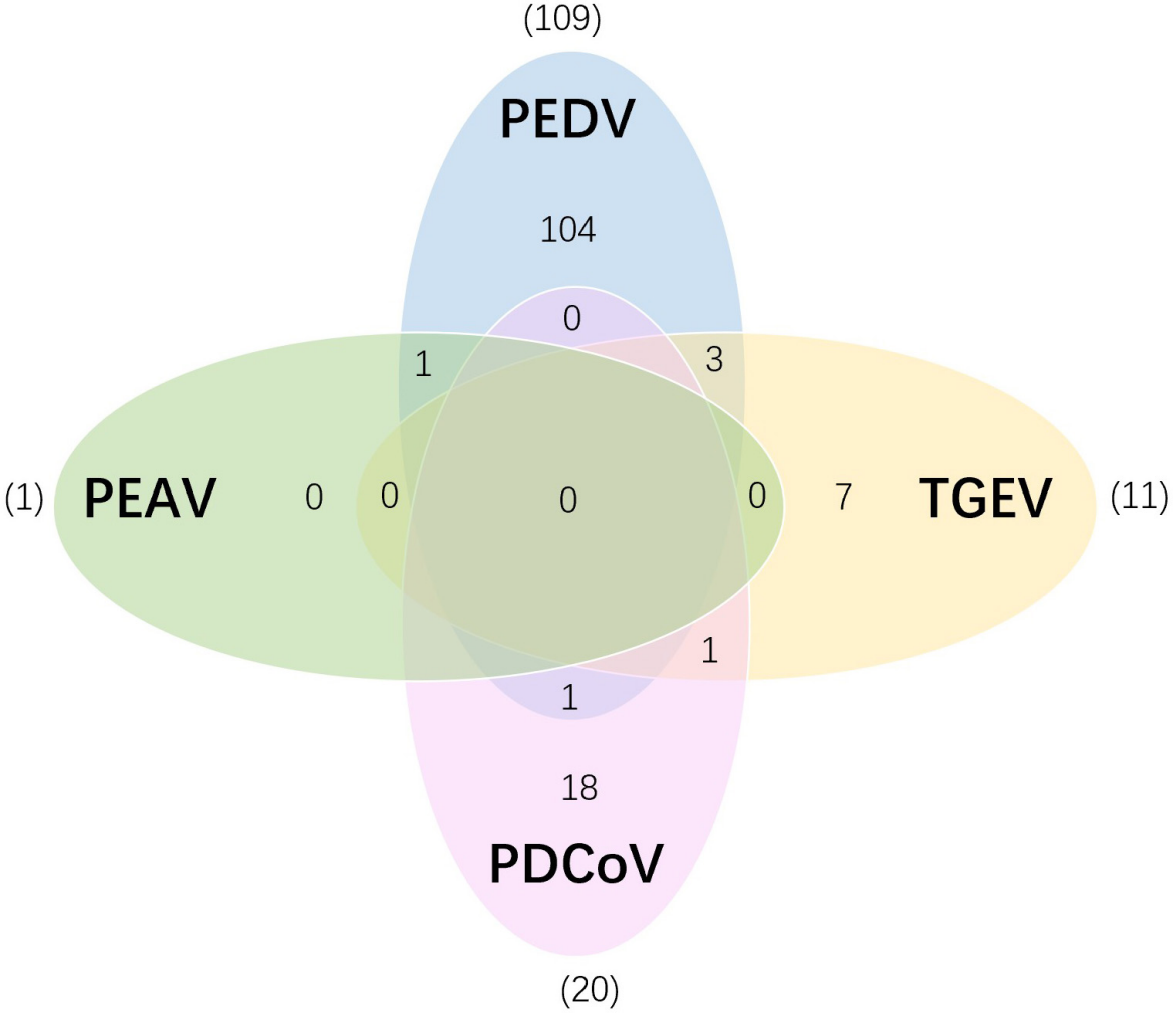
Co-infection analysis of detected enteric coronaviruses. The Venn diagram shows the number of clinical samples infected with a single or multiple enteric coronaviruses. The overlapping numbers (1 and 3) represent the number of co-infection (e.g., 3 represents PEDV + TGEV co-infection).

The applicability of our quadruple RT-qPCR method was evaluated by testing a total of 231 clinical samples including intestinal tissue, fecal samples, and anal swabs collected from different regions in Fujian. Results showed that the total positive rate of the four enteric coronaviruses was 64.93% (150/231), with the positive rates of PEDV, PEAV, PDCoV, and TGEV were 51.52% (109/231), 1.73% (1/231), 8.66% (20/231), and 4.76% (11/231), respectively (Table 4); the co-infection rates of PEDV and PEAV, PEDV and PDCoV, PEDV and TGEV, and TGEV and PDCoV were 0.43% (1/231), 0.43% (1/231), 1.3% (3/231), and 0.43% (1/231), respectively (Figure 4). Above samples were also tested using the commercialized RT-qPCR kits, and the results were consistent with the results tested using the method established in this study, with a 100% agreement rate, indicating the high reliability and accuracy of this method in clinical sample testing.

**Table 4:** Positive rates detected from the clinical samples.

**Pathogens**	**Positive**	**Percentage (%)**
PEDV	109	51.52
PEAV	1	1.73
PDCoV	20	8.66
TGEV	11	4.76
Total	231	64.93

## 4 Discussion

Piglet enteric diseases caused by viral infection pose a severe threat to the pig industry worldwide, leading to a high mortality in piglets and significant economic losses (5). A major challenge in managing these diseases is the frequent co-infection with multiple diarrheal viruses, which present highly similar clinical symptoms, thereby significantly complicating disease prevention and control. Among the main pathogens responsible for piglet diarrhea are PECs, including PEDV, PEAV, PDCoV, and TGEV. Infection with any of these viruses can lead to severe diarrhea, vomiting, dehydration, and high mortality in piglets (17). Given the high similarity in the clinical symptoms and pathological lesions caused by these coronaviruses, it is critical to establish a rapid, stable, and sensitive method for their simultaneous detection and discrimination. Molecular diagnostic techniques for detecting PECs have been developed such as loop-mediated isothermal amplification (LAMP) and microfluidics (18). The advantages of LAMP include visualization, rapidity, high sensitivity, and isothermal amplification, making it highly suitable for on-site detection (18, 19). However, it is prone to frequent false positives due to aerosol contamination, a problem that is difficult to resolve (20). Microfluidic chips represent an innovative diagnostic platform that enables rapid sample analysis by utilizing microliter-scale reaction volumes (21). However, the complex manufacturing process and the poor batch consistency make high repeatability difficult to achieve. Moreover, neither technique can simultaneously identify four PECs.

The Real-time qPCR technique is a detection tool known for its rapidity, high specificity, high sensitivity, and excellent reproducibility in virus detection (22, 23). In recent years, the application of multiplex qPCR has made great progress for detecting porcine intestinal coronaviruses, and many research groups have improved the detection efficiency by optimizing target genes and reaction systems (24–26). Initially, dual fluorescence qPCR methods achieved precise detection of common diarrheal coronaviruses (22). However, a limitation of these methods is their inability to detect newly emerged viruses such as PDCoV and PEAV, a capability that is increasingly necessary given the prevalence of co-infections. With the emergence of novel coronaviruses such as PDCoV and PEAV, multiplex RT-qPCR technique has gradually become a research hotspot. Several multiplex qPCR methods established for the differential diagnoses of PECs have been published in recent years, with primers and probes designed for different conserved regions of these viruses (10, 13–15, 24–26).

In this study, we established a highly specific multiplex real-time RT-qPCR assay for the detection and differential diagnosis of swine enteric coronaviruses. Specific TaqMan probes were designed to target the highly conserved regions of PEDV, PEAV, PDCoV, and TGEV. Among the open reading frames (ORFs) and four structural protein genes [M, N, S (spike), and E (envelope)] of coronaviruses, the M and N genes are more conserved and less homologous among the four porcine enteric coronaviruses than the S and E genes (13, 27). Therefore, M genes of three viruses (PEDV, PEAV, and PDCoV) and N gene of TGEV were selected as targets. The results demonstrated that each primer and probe set was highly specific, detecting only its intended target without cross-reaction with other viruses. Although the sensitivity of our multiplex qPCR assay is lower than that of singleplex qPCR, probably due to competition for primers, probes, templates, and reagents (13, 28), our clinical sample testing results demonstrated that this achieved sensitivity is sufficient for field applications for samples with typical viral loads. The established multiplex RT-qPCR method successfully identified all four coronaviruses and exhibited high stability, with a coefficient of variation between 0.3 and 1.0%. Application of this method to clinical samples revealed a high infection rate and a high frequency of co-infections. This common phenomenon highlights the clinical necessity and significance of developing a multiplex RT-qPCR for rapid diagnosis. The results demonstrated that PEDV was the predominant pathogen responsible for swine diarrhea on pig farms in Fujian, which is consistent with other studies (29, 30). Indeed, PEDV is the primary cause of piglet diarrhea today, complicating disease prevention and thus inflicting severe economic losses on the Chinese swine industry (31). Furthermore, co-infections with swine enteric coronaviruses were common in some pig farms. Co-infection may cause recombination between viruses and promote the evolution of non-pathogenic enteric viruses into highly pathogenic viruses (24). In addition, to validate the results, all samples were tested by the reference method, and the results showed 100% agreement between the two methods. Future studies employing this method should include samples from other regions to strengthen its epidemiological relevance. Furthermore, due to the sensitivity limitation of this method, future validation should be conducted on samples with low quality or low viral loads.

In summary, we established a quadruple RT-qPCR method with high specificity, sensitivity and stability for the simultaneous detection of four porcine diarrheal coronaviruses: PEDV, PEAV, PDCoV, and TGEV. This method provides a reliable tool for the synchronized monitoring of multiple pathogens on pig farms and offers a robust system for the early warning of diarrheal diseases.

## Data Availability

The original contributions presented in the study are included in the article/supplementary material, further inquiries can be directed to the corresponding author.
